# Design and validation of a multiplex PCR protocol for microsatellite typing of *Candida parapsilosis* sensu stricto isolates

**DOI:** 10.1186/s12864-018-5065-3

**Published:** 2018-09-29

**Authors:** Camino Trobajo-Sanmartín, Guillermo Ezpeleta, Célia Pais, Elena Eraso, Guillermo Quindós

**Affiliations:** 1 Laboratorio de Micología Médica, Departamento de Inmunología, Microbiología y Parasitología, UFI11/25 (Microbios y Salud), Facultad de Medicina y Enfermería, Universidad del País Vasco/Euskal Herriko Unibertsitatea (UPV/EHU), Apartado 699, E-48080 Bilbao, Spain; 2 Departamento de Medicina Preventiva y Salud Pública, Facultad de Medicina y Enfermería, Universidad del País Vasco/Euskal Herriko Unibertsitatea (UPV/EHU), Bilbao, Spain; 3 Servicio de Medicina Preventiva e Higiene Hospitalaria, Complejo Hospitalario de Navarra, Pamplona, Spain; 4 Centro de Biologia Molecular e Ambiental (CBMA), Departamento de Biologia, Universidade do Minho, Braga, Portugal

**Keywords:** *Candida parapsilosis* sensu stricto, Genotyping technique, Microsatellites repeats, Multiplex PCR, Specificity, Reproducibility of results

## Abstract

**Background:**

Analysis of polymorphic microsatellite markers (STR) is a helpful genotyping technique to differentiate *Candida parapsilosis* sensu stricto isolates. The aim of this study is to develop and perform an initial validation of an alternative protocol for the reliable and accurate microsatellite genotyping of *C. parapsilosis* sensu stricto isolates using high-throughput multiplex PCR. To achieve this, the results obtained using the new protocol were compared to the ones obtained using a previously described reference method. To that end, diagnostic accuracy, informativeness and discrimination parameters were estimated.

**Results:**

Our results showed good concordance between both methods (Kappa index: 0.920), leading to a high sensitivity (1; CI(95%) (0.991–1)) and specificity (1; CI(95%) (0.772–1)) after the validation of the new protocol. Moreover, the electropherograms profiles obtained with the new PCR scheme showed a high signal to noise ratio (SNR).

**Conclusions:**

The new multiplex protocol is valuable for the differentiation of *C. parapsilosis* sensu stricto, with direct clinical applications. Besides, the new protocol represents a shortening the hands-on time, reducing the sample manipulation (dismissing the possibility of cross-contamination), maintaining the quality of the results (when compared to the ones obtained with the reference method), and helping to the standardization and simplification of the genotyping scheme.

## Background

In 2005, Tavanti et al. proposed that the fungus *Candida parapsilosis* could be considered as a genetically related species complex which includes *C. parapsilosis* sensu stricto, *Candida metapsilosis* and *Candida orthopsilosis* [1], being *C. parapsilosis* sensu stricto the most commonly isolated. However, *C. parapsilosis* sensu stricto is not a homogeneous species, and therefore, accurate and reliable typing methods are necessary for a better knowledge of this species [1–3]. These typing methods have been used for identifying the sources of infection, the chain of transmission and for determining the dissemination of specific strains in the medical environment [4].

Last decades technological innovations allowed the extensive use of microsatellites or Single Sequence Repeats (SSR) in plant and eukaryote genetics studies, using different genotyping approaches ranging from low to high throughput ones, not only in genetic research but also with interesting applications in clinical practice. In fact, microsatellite typing has been described to study genetic relatedness among colonizing and infective strains from diverse geographical locations or even the relatedness of *C. parapsilosis* isolated from different clinical sources, such as blood or catheters, and from medical or surgical wards [2, 4, 5]. Currently, the microsatellite typing method described by Sabino et al. [5] is one of the reference techniques to show genetic relatedness among different clinical isolates of *C. parapsilosis* sensu stricto.

This issue aroused an explosion of alternative protocols to standard procedures giving a large number of genotyping schemes but with no extensive validation by comparing them to the existing ones [6–8]. This work aimed to develop and perform an initial validation of an alternative protocol for the reliable and accurate microsatellite genotyping of *Candida parapsilosis* sensu stricto isolates using high-throughput multiplex PCR.

## Methods

### Microorganisms

Thirty-three *C. parapsilosis* sensu stricto blood isolates retrospectively collected during 2010 to 2015 using a convenience sample of 33 patients suffering from invasive candidemia during their hospital stay at La Fe University Hospital (Valencia, Spain). In addition to those clinical isolates, *C. parapsilosis* sensu stricto ATCC 22019, and ATCC MYA-4646 (CDC317) obtained from the American Type Culture Collection (ATCC, Manassas, VA, USA) were used as positive controls. Moreover, *C. orthopsilosis* ATCC 96139, *C. metapsilosis* ATCC 96143, *Candida albicans* ATCC 90028, *Candida africana* ATCC MYA-2669, *Candida dubliniensis* NCPF 3949, *Candida glabrata* ATCC 90030, *Candida bracarensis* NCYC 3133, *Candida nivariensis* CBS 9984, *Candida tropicalis* NCPF 311, *Candida krusei* ATCC 6258, *Candida guilliermondii* NCPF 3099 and *Lodderomyces elongiosporus* ISA1308 (NCPF, National Collection of Pathogenic Fungi, UK; NCYC, National Collection of Yeast Cultures, UK; CBS, Centraalbureau voor Schimmelcultures, Netherland and ISA, Instituto Superior de Agronomía, Portugal) were included as subrogate negative controls to assess the specificity of the multiplex PCR protocol. Besides, PCR grade water (Roche Diagnostics, Germany) was used as negative control for all PCR tests performed.

### Culture, isolation and identification

All isolates were plated onto Sabouraud dextrose agar (Difco, USA) and incubated at 37 °C for 24 h. Presumptive identification was performed considering colony morphology and color on ChromID Candida (bioMérieux, France) and Candida chromogenic agar (CONDA, Spain) agars and subsequently confirmed using the API 32C (bioMérieux) auxanogram according to manufacturers. DNA was extracted using the UltraClean® Microbial DNA Isolation Kit (MoBio, USA) following the recommendations of the manufacturers. Definitive identification was reached either by amplification of a short portion of the *SADH* gene using a conventional RFLP-PCR protocol, as previously described [1, 9] or by ITS sequencing when the obtained RFLP-PCR profile from the former technique was inconclusive.

### Optimization of multiplex PCR scheme conditions

This step was only performed using the control strains and each prepared multiplex reaction was tested under different primer concentrations (ranging from 0.2 to 0.5 μM) to ensure the best PCR performance. After this step, another multiplex reaction was tested at different final DNA concentrations (ranging from 10 to 30 ng) to establish the best amount of yeast genomic DNA needed. Finally, the addition of bovine serum albumin (purified BSA, 100X) (New England Biolabs, USA) to the PCR mastermix to enhance the reaction efficacy was evaluated.

#### Assay for establishing differences in the polymerase activity

Based on previous reports of polymerase inefficiency of prominent slippage phenomena under certain conditions [10, 11], we performed a short experiment to evaluate the performance of three different specially designed for multiplex PCR amplification commercial polymerases and their respective mastermixes. To that end, each reference strain was tested in parallel under same amplification conditions using the mentioned mastermixes and its correspondent DNA polymerase. The PCR mastermixes included in the assay were TaKaRa Ex TaqTM Hot Start Version (Takara Bio Inc., Japan), KAPA2G Fast Multiplex PCR kit (Kapa Biosystems, UK) and AmpliTaq Gold® DNA Polymerase (Applied Biosystems Inc., USA).

#### Modified multiplex microsatellite PCR amplification

All *C. parapsilosis* sensu stricto were genotyped using CP1, CP4, CP6 and B5 microsatellite markers previously described by Sabino et al. [5]. Because of the rather low annealing temperatures of the designed primers, a multiplex touchdown PCR protocol was chosen to prevent (or minimize) the appearance of unspecific (or non-desirable) PCR products. The amplification reaction had a final volume of 25 μl containing 15 ng of yeast genomic DNA, 1X PCR buffer, 1X BSA (100X), 1.25 U of DNA polymerase, 1.5 mM MgCl_2_, 0.4 μM of each primer, and 0.2 mM deoxynucleoside triphosphates (dNTPs) and the amplification touchdown PCR protocol was performed in a C 1000TM Thermal Cycler (Bio-Rad, USA).

**Fig. 1:**
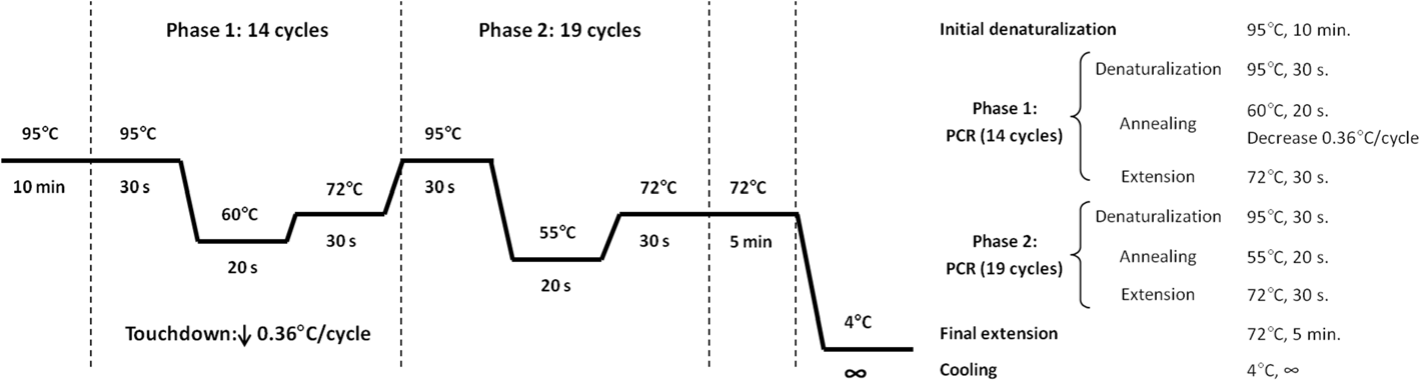
Summary of the touchdown PCR protocol

Briefly, PCR protocol had two differentiated phases. In the first step, annealing temperature of 60 °C gradually decreased 0.35 °C per cycle until it reached 55 °C. The second phase was the same as the latter except for three minor modifications, which were a slight increase in the number of cycles (from 14 to 19), a fixed annealing temperature of 55 °C (see Fig. 1 for more details).

### Fragment size determination

Once the PCR protocol was optimized, 33 *C. parapsilosis* sensu stricto blood isolates were genotyped using the multiplex scheme proposed in this work and the singleplex PCR protocol described by Sabino et al. [5]. For each PCR product size determination, each tested allele forward primer was labeled with a different fluorochrome: 5′ 6-Fluorescein (56-FAM) for CP1, 5′ MAX (NHS Ester) (5MAXN) for CP4, 5′ 5-TAMRA™ (Azide) (55-TAMK) for CP6, and finally 5′ Rhodamine Red™-X (NHS Ester) (5RhoR-XN) for B5 (IDT, Belgium). One microliter of each obtained amplification product was mixed with 8.6 μl of Hi-Di formamide and 0.4 μl of the internal size standard (GenScan™ 500 LIZ® Size Standard; Applied Biosystems). This mixture was heated for 5 min at 95 °C and immediately cooled to 4 °C to ensure DNA detachment. After denaturalization, the samples were run on an ABI PRISM® 3130xl Genetic Analyzer (Applied Biosystems) and the final size of the obtained PCR products was determined using the PeakScanner software (version 1.0). The same software was used for the estimation of the number of repeats in each processed allele by direct comparison of the relative size of the clinical isolate to the defined for *C. parapsilosis* sensu stricto reference strains.

### Genotype definition and data analysis

The microsatellite genotypes were defined on the unique combination of alleles obtained for the four loci analyzed and considering that the size differences observed at one or more loci defined different genotypes.

The identification of similarities between genotypes was achieved by the constructions of a minimum spanning tree using R statistical software (v.3.1.0). Besides, to represent the relationship between all the *C. parapsilosis* sensu stricto genotypes obtained, a phylogenetic tree was performed using the POPTREE software. Basically, the phylogenetic tree was inferred from the allele frequency data obtained from the studied samples, was performed using the neighbor-joining method or the unweighted pair-group method with arithmetic mean (UPGMA). Additionally, a bootstrap test was implemented for evaluating the robustness of the results [12].

Besides, we also calculated other parameters of each microsatellite marker considered in this study which are linked to the microsatellite informativeness content and their discrimination power such as the polymorphic information content (PIC), the Simpson index, the heterozygosity and the entropy [13, 14].

Finally, an estimation of sensibility, specificity, and the Kappa index was performed to estimate not only the diagnostic characteristics of each microsatellite detection protocol but also the agreement among the results obtained after the microsatellite amplification using each compared PCR protocol. All the statistical procedures were performed using the Stata(R) and R statistical software (v. 12 and 3.1.0 respectively). The associations between categorical variables were studied using a chi-squared test or Fisher’s exact test when necessary.

### Ethical issues

This study does not involve human participants, human data or human tissue. The authors solely used *C. parapsilosis* sensu stricto strains from different repositories or collections to fulfill the objectives of the study. Although some of the strains used for validation came from a clinical origin, no processing of primary samples was made during the experimental work and therefore, the need for ethics approval and consent to participate was unnecessary according to the Spanish Biomedical Research Law and other European Union regulations. However, a formal approval was asked to the Ethical and Research Committee of the University of the Basque Country to ensure that all the issue research was in accordance with the legal and ethical requests prior to its beginning (Ethics Committee of the Universidad del País Vasco/Euskal Herriko Unibertsitatea UPV/EHU, Bilbao, Spain, reference number CEIAB M30_2015_248).

## Results

### Redesign and optimization of original PCR protocol

Despite the several approaches implemented along with the literature to establish a successful microsatellite based genotyping scheme, we focused on the optimization and restructuring of the original PCR protocol proposed by Sabino and coworkers [5] converting it from a singleplex approach to a multiplex one, avoiding the redesign of the initial primer pairs.

Table 1 summarizes the main the characteristics, advantages and disadvantages of different successful *C. parapsilosis* sensu stricto microsatellite genotyping protocols published along the literature compared to the one proposed in our study. The optimization and redesign strategy mentioned earlier implied the evaluation and subsequent election of the two cornerstones of the PCR reaction: the polymerase and primer concentration.

**Table 1:** Main characteristics, pros and cons between four *C. parapsilosis* sensu stricto microsatellite genotyping protocols published in the literature compared and the one described in this work (*N* = 5)

Characteristic	Sabino et al. [5]	Diab-Elschahawi et al. [6]	Reiss et al. [7]	Vaz et al. [8]	Trobajo-Sanmartín et al. (This study)
PCR scheme used	Singleplex^b^	Multiplex	Multiplex	Multiplex	Multiplex
Redesign of the original primers	NA	Yes	Yes	Yes	No
Number of primers pairs used for each PCR reaction	1	3	1	4	4
Total number of PCR reactions needed for a complete STR analysis	3	2	5	1	1
Number of dyes used in each genotyping reaction	3	3	1	3	4
Evaluation of the sensitivity and specificity^a^	NA	No	No	No	Yes
Evaluation of the microsatellite informativeness parameters	Yes	Unknown^c^	Unknown^c^	Unknown^b^	Yes
Total time elapsed to obtained results (in minutes)	138	79	197	138	79
Approximate cost^d^ estimated of the primer pairs used in each protocol (in euros)	5,60	15,72	7,00	9,26	12,76
Approximate total cost^d^ estimated for the complete STR analysis of one sample (in euros)	15,58	22,53	20,77	14,00	17,45

*NA* Not applicable

^a^Taking the original protocol published by Sabino and coworkers as the gold standard

^b^One of the four reactions could be multiplexed using three different dyes

^c^Probably the authors did the evaluation of the microsatellite informativeness parameters, but there is no clear reference to that subject in their manuscript

^d^Prices are referred to Spain. We included the DNA extraction, AmpliTaq Gold® PCR kit with magnesium chloride and PCR buffer, PCR plate (96-well) and primer prices to estimate the total cost per sample analyzed using each protocol

Table 2 summarizes the results obtained during the modified protocol optimization including the sensitivity, specificity and positive predictive value for each polymerase enzyme tested in this study. Our results pointed out that the use of different sort of polymerases could affect to the PCR result. In our experience, the AmpliTaq® Gold polymerase was the only one of those tested that showed values for both sensitivity and specificity equal to 100%.

**Table 2:** Sensitivity, specificity and predictive values of the multiplex protocol using different polymerases (*N* = 47)

Polymerase	FP	FN	TP	TN	Sensitivity (CI 95%)	Specificity (CI 95%)	PPV (CI 95%)	NPV (CI 95%)
TaKaRa® Ex Taq Hot Start Version	12	0	35	0	100%	0%	74.47%	NA
					(90.1%. 100%)	(0.0%. 26.5%)	(59.7%. 86.1%)	(NA)
KAPA 2G Fast Multiplex PCR kit	12	0	35	0	100%	0%	74.47%	NA
					(90.1%. 100%)	(0.0%. 26.5%)	(59.7%. 86.1%)	(NA)
AmpliTaq® Gold DNA Polymerase	0	0	35	12	100%	100%	100%	100%
					(90.1%. 100%)	(73.5%. 100%)	(90.1%. 100%)	(73.5%. 100%)

*FP* False positive results, *FN* False negative results, *TP* True positive results, *TN* True negative results, *PPV* Positive predictive value, *NPV* Negative predictive value, *CI 95* 95% confidence interval, *NA* Not available

Furthermore, we found that there were false positive results (non-specific bands) when we used the KAPA2G and Takara mastermixes. Besides, based on our findings, among all the concentrations tested, we found that the 0.4 μM final concentration of each allele primer pair lead to the best PCR results. Using this primer concentration, all PCR products obtained by the multiplex protocol showed the same intensity.

### Samples genotyping results

Table 3 reflects the microsatellite genotyping results for the four loci considered under the two conditions tested in our work. The obtained microsatellite typing results using the protocol described by Sabino et al. [5] compared to the ones using the multiplex PCR protocol proposed in our work, both were identical. This issue lead a high sensitivity (1; CI(95%) (0.991–1)) and specificity (1; CI(95%) (0.772–1)) values during the validation step of the new protocol. Furthermore, both techniques showed no amplification in any of the negative control strains (even close-related species such as *L. elongiosporus*) included in this study. Besides, the ATCC 22019 positive control strain gave the same profile described in the literature after microsatellite fragment analysis (Table 3) [2, 15].

**Table 3:** Microsatellite fragment analysis after different PCR schemes (*N* = 35)

Strain	Microsatellite (bp) Sabino’s protocol				Microsatellite (bp) This study			
	CP1	CP4	CP6	B5	CP1	CP4	CP6	B5
MYA-4646	243/243	308/327	292/295	154/154	242/242	307/327	291/294	154/154
ATCC 22019	244/250	306/306	292/292	132/132	242/247	307/307	293/293	136/136
429	239/242	312/358	250/250	139/139	239/242	310/358	250/250	139/139
431	236/242	309/309	265/282	133/138	236/242	307/307	264/281	133/139
476	222/242	308/370	265/265	133/133	236/242	307/307	264/264	139/139
477	239/242	327/327	264/264	135/135	239/242	326/326	264/264	134/134
480	222/242	364/370	268/271	120/135	222/242	362/370	267/270	120/134
482	242/271	352/370	291/294	133/137	242/270	351/370	290/293	132/136
486	222/242	373/373	306/306	135/135	222/242	372/372	305/305	134/134
489	239/239	308/308	259/259	154/154	239/239	307/307	258/258	152/152
491	242/271	370/370	269/291	132/132	242/271	369/369	290/290	132/132
499	242/242	361/364	267/270	120/135	242/242	360/363	267/270	119/134
504	237/243	309/309	265/283	133/139	236/242	307/307	264/282	132/138
509	239/242	394/397	270/302	133/145	239/242	390/390	270/302	132/144
512	216/222	373/373	305/308	135/135	216/222	372/372	305/308	134/134
514	222/242	372/372	309/309	135/135	222/242	372/372	308/308	134/134
517	217/223	373/373	306/309	135/135	216/222	372/372	305/308	134/134
521	222/242	370/370	271/320	135/135	222/242	369/369	270/319	134/134
522	222/242	370/370	320/320	135/135	222/242	370/370	320/320	134/134
534	239/242	379/379	288/294	135/135	239/242	379/379	288/294	125/134
536	222/242	370/385	273/320	135/135	222/242	369/384	273/320	134/134
542	239/242	324/343	253/253	163/163	239/242	323/343	253/253	163/163
543	239/239	355/355	273/273	111/111	239/239	354/354	273/273	110/110
547	239/242	379/379	250/317	135/135	239/242	379/379	250/250	134/134
565	239/242	352/352	273/308	111/111	239/242	351/351	273/308	110/110
568	222/242	373/373	305/308	135/135	222/242	372/372	305/308	134/134
569	222/242	370/370	318/321	135/135	222/242	369/369	317/320	134/134
588	222/242	370/370	320/320	135/135	222/242	369/369	320/320	135/135
591	222/242	370/370	276/320	135/135	222/242	369/369	276/319	135/135
592	239/242	400/420	270/302	133/145	239/242	400/420	270/302	133/145
593	219/259	308/308	267/288	153/155	219/259	307/307	267/287	153/156
595	222/242	370/370	270/320	135/135	222/242	369/369	270/320	135/135
596	242/242	361/364	267/270	120/135	242/242	360/363	267/270	120/135
599	236/242	308/308	264/264	139/139	236/242	307/307	264/264	139/139
600	242/242	361/364	267/270	120/135	242/242	360/363	267/270	120/135

**Fig. 2:**
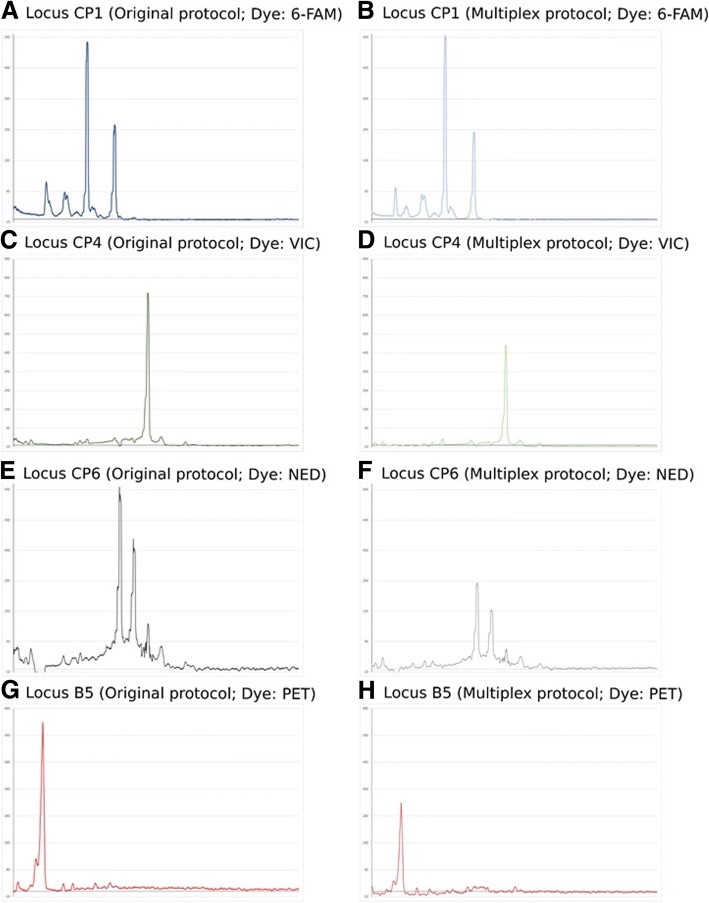
Example of the electropherograms obtained after genotyping the one of the clinical isolates *Candida parapsilosis* sensu stricto (clinical isolate 593) using the original simplex protocol described by Sabino and coworkers (**a**, **c**, **e**, and **g**) and the multiplex one used in this study (**b**, **d**, **f**, and **h**)

Despite these excellent results, the electropherograms obtained frequently showed low-intensity non-specific bands, which were considered as stutter bands due to the polymerase slippage during the PCR (Fig. 2). However, the small amplitude of these artifacts did not interfere with the correct identification of the fragment size and subsequently had no impact on the new protocol specificity.

Besides, the similar signal-to-noise ratio of the resulting electropherograms was recorded when compared the new proposed touchdown PCR scheme to the original protocol described by Sabino et al. in 2010 or other slight modifications to that one [2, 4, 8, 16].

**Fig. 3:**
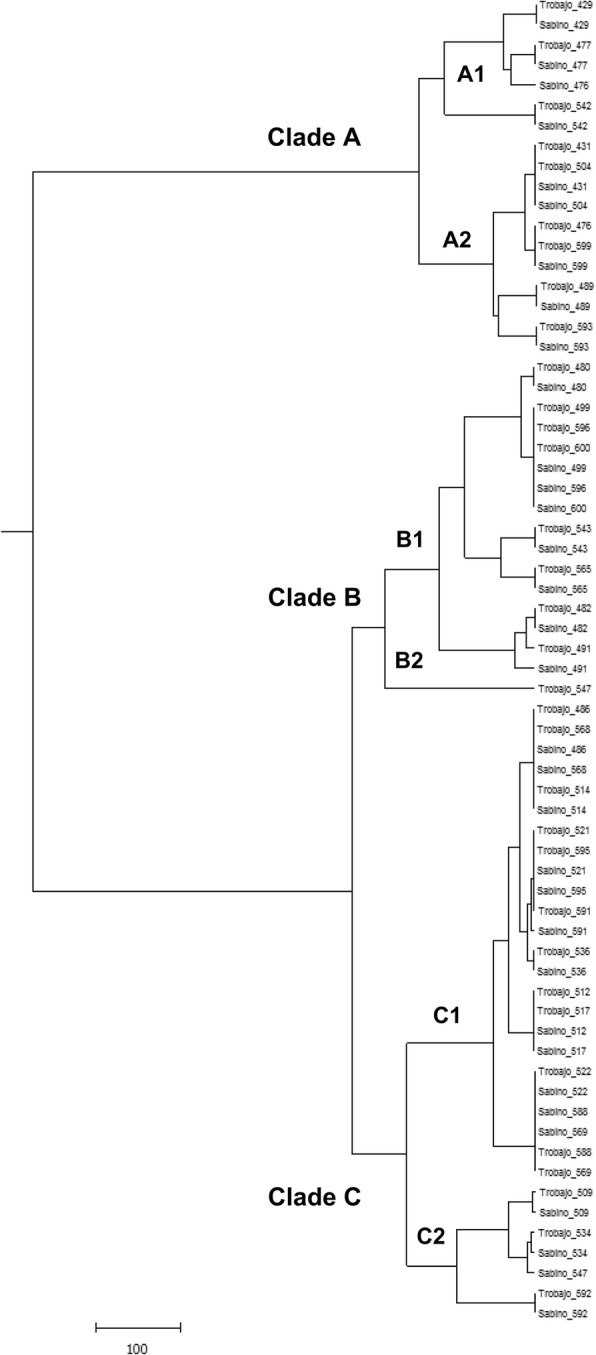
An unweighted pair group method dendrogram based on the microsatellite genotypes identified from the clinical isolates *Candida parapsilosis* sensu stricto (*N* = 33) analyzed in this study

To obtain a graphical view of the results mentioned above, we performed a dendrogram based on the microsatellite genotypes identified from the clinical isolates *C. parapsilosis* sensu stricto analyzed using both protocols. This dendrogram is represented in Fig. 3.

#### Estimation of the information contained in the regions examined

The obtained estimates of the informativeness parameters investigated are summarized in Table 4. Regarding the observed allele heterozygosis of the analyzed *C. parapsilosis* sensu stricto strains, our results revealed several differences among the analyzed loci. The heterozygosis percentages ranged from 84.85% for locus CP1 to 27.27% for locus CP4. However, the heterozygosis rates observed for locus CP6 and locus B5 were 60.61% and 33.33%, respectively. The discrimination power of each considered allele was concordant with those previously published by Sabino et al. [5]. The Simpson index oscillated from 0.702 for CP1 to 0.925 for CP6 marker, which means that the CP1 marker achieved the lowest discrimination power.

**Table 4:** Discrimination and information parameters for each microsatellite markers. The Simpson index and the observed heterozygosis obtained in the original procedure by Sabino et al. [5] are also included

Parameters	CP1	CP4	CP6	B5	Multiplex	Study
Polymorphic information content (PIC)	0.692	0.870	0.942	0.802	0.957	This study (2016)
Simpson Index	0.724	0.879	0.944	0.816	0.959	
Observed heterozygosis	0.723	0.273	0.606	0.333	0.958	
Unbiased estimation of heterozygosis	0.724	0.880	0.945	0.816	0.960	
Entropy (in bits)	2.450	3.647	4.430	3.179	4.688	
Simpson Index	0.850	0.890	0.960	0.860	0.990	Sabino et al. (2010) [5]
Observed heterozygosis	0.733	0.214	0.523	0.300	–	

### Concordance analysis

Table 5 summarizes the concordance between the improved multiplex protocol and the reference genotyping technique using the direct concordance and the Kappa indices, being both greater than 80%. According to the literature, these results suggest that there is a high concordance level among both genotyping schemes [17].

**Table 5:** Direct concordance and Kappa indices of the multiplex PCR touchdown protocol

Multiplex PCR approach genotyping technique (this study)	Original genotyping technique [5]						
	Clade A1	Clade A2	Clade B1	Clade B2	Clade C1	Clade C2	Total
Clade A1	3	0	0	0	0	0	3
Clade A2	1	5	0	0	0	0	6
Clade B1	0	0	8	0	0	0	8
Clade B2	0	0	0	0	0	1	1
Clade C1	0	0	0	0	12	0	12
Clade C2	0	0	0	0	0	3	3
Total	4	5	8	0	12	4	33
Agreement	Expected agreement	Kappa index		Std. Error of Kappa index		Z	*p*-value
0.939	0.240	0.920		0.0917		10.04	< 0.001

## Discussion

Several methods, such as isoenzyme analysis, random amplified polymorphic DNA (RAPD), restriction fragment length polymorphism (RFLP) and multilocus sequence typing (MLST), have been described for *Candida* isolates typing [1, 18, 19]. However, the discriminatory power of some of these typing methods for differentiating *C. parapsilosis* isolates is rather small and many isolates are indistinguishable [3]. In recent days, matrix-assisted laser desorption/ionization time-of-flight mass spectrometry (MALDI TOF-MS) and the analysis of polymorphic microsatellite regions have been described as useful and high discriminatory power techniques for further differentiation of *C. parapsilosis* sensu stricto isolates [3, 20]. However, it seems that MALDI-TOF MS-based typing does not fully correlate with other DNA-based genotyping methods leading to different dendrogram profiles when using protein-based or DNA-based techniques and moderate concordance values between those techniques [21]. Therefore, though MALDI TOF-MS is a reliable technique for identifying isolates at species-level, perhaps more studies are needed to assess its role in fungal genotyping [21, 22].

Until recently, microsatellite genotyping is a rather time-consuming technique, because every microsatellite marker must be processed alone. Up to our knowledge, no multiplex PCR protocol following the original scheme proposed by Sabino et al. [5] has been described to that end along with the literature. Recently, Diab-Elschahawi et al. [6] published a PCR protocol using a multiplex approach for CP1, CP4 and CP6 markers redesigning the primers proposed in the original work by Sabino et al. [5, 6]. The disparities between the annealing temperatures of the original primers designed by Sabino and coworkers [5] difficult the amplification of all the loci at the same time, and therefore, other approaches such as primer redesign are necessary. Despite the success of these redesigned primer protocols, we focused a different solution based on optimization and redesign of the PCR protocol (from a singleplex approach to a multiplex one) avoiding the redesign of the initial primer pairs. This solution gave comparable results to the original approach published by Sabino et al. [5] during the validation step with high sensitivity and specificity.

Based on our results, there are several crucial points to consider before getting satisfactory results, being the appropriate polymerase election the most important one when a multiplex PCR protocol is used. Although all the PCR mastermixes tested in our work were explicitly fabricated to operate under their best conditions using multiplex PCR protocol, KAPA2G and Takara mastermixes, showed lack of specificity, conditioning their future use in multiplex PCR based *C. parapsilosis* sensu stricto genotyping protocols. The most reliable explanation of the observed results is that the three mastermixes tested had different polymerases in their composition, being the AmpliTaq Gold® the most suitable one to carry out *C. parapsilosis* sensu stricto microsatellite genotyping using this multiplex PCR scheme.

A total of 35 samples were genotyped to validate the utility of our method in contrast to the one described by Sabino and coworkers. Though our results were concordant with those published previously, we could see slight differences in the estimation of the Simpson index and the observed heterozygosis among Sabino’s original data and ours, probably explained because of the differences in the total sample number of strains analyzed in each work.

Finally, the high-quality profiles of the electropherograms obtained using the new multiplex protocol are due to the adoption of a touchdown PCR strategy which improves the profile analysis and prevents misclassification. In a recent review, such schemes are described as a suitable option to increase the specificity of the obtained PCR products without losing sensitivity [23].

There are some limitations in our study such as the small number of strains analyzed in this study and the fact that all of them were isolated from the same clinical source (blood). This issue has probably an impact on the precision of the confidence intervals and the generalization of our informativeness parameters estimates. However, the consistency of our results with those published in the literature suggesting that the possibility of bias is rare.

Despite these limitations, our validation results support that the new protocol seems to be as accurate and reliable as the original one. However, it represents a significant decrease in the turnaround time necessary to get accurate genotyping results compared to other approaches published along with the literature. The main disadvantage the new protocol is that it is slightly more expensive than the original technique in case we use primers labeled with different fluorophores. This limitation could be overcome by using the same fluorophore for those primers targeting loci that have very different sizes (such as CP6 and B5), decreasing the total cost of the technique and increasing its cost-effectiveness if the researchers decide to adopt the latter option.

## Conclusions

In conclusion, this new protocol is a valuable tool for the differentiation of *C. parapsilosis* sensu stricto isolates, with direct applications to clinical practice and infection control procedures (for example, nosocomial outbreaks). Besides, our protocol helps the standardization and simplification of the existing microsatellite typing systems, improving the quality of data, the sample hands-on time and lab turnaround time to get accurate genotyping results for further clinical or infection control epidemiological studies.

## Abbreviations

55-TAMK: TAMRA-tetramethyl-6-rhodamine
56-FAM: 56-carboxyfluorescein
5MAXN: Max 557 NHS ester
5RhoR-XN: Rhodamine Red-X
ATCC: American type culture collection
bp: Base pair
BSA: Bovine serum albumin
CBS: Centraalbureau voor Schimmelcultures
CI: Confidence interval
DNA: Deoxyribonucleic acid
dNTPs: Deoxynucleotide triphosphates
FN: False negative
FP: False positive
ISA: Instituto Superior de Agronomía
MALDI TOF-MS: Matrix-assisted laser desorption/ionization time-of-flight mass spectrometry
MLST: Multilocus sequence typing
NCPF: National Collection of Pathogenic Fungi
NCYC: National Collection of Yeast Cultures
NPV: Negative predictive value
PCR: Polymerase chain reaction
PIC: Polymorphic information content
PPV: Positive predictive values
RAPD: Random amplified polymorphic DNA
RFLP: Restriction fragment length polymorphism
SADH: Secondary alcohol dehydrogenase
SNR: Signal to noise ratio
SSR: Single sequence repeats
STR: Short tandem repeat
TN: True negative
TP: True positive
U: Units
UPGMA: Unweighted pair-group method with arithmetic mean

## SI units

°C: Celsius degrees
h: Hour
min: Minute
mM: Millimolar
ng: Nanograms
s: Second
μl: Microliters
μM: Micromolar
